# GLA is associated with ESCC progression and chemotherapy response via DNA damage repair–related pathways

**DOI:** 10.3389/fonc.2026.1900889

**Published:** 2026-07-15

**Authors:** Ke Chen, Qinsong Yang, Chen Fang, Weiran Zhang, Yu Feng, Haitao Ma

**Affiliations:** 1 Department of Thoracic Surgery, The First Affiliated Hospital of Soochow University, Suzhou, China; 2 Department of Thoracic and Cardiovascular Surgery, Fourth Affiliated Hospital of Soochow University, Suzhou, China

**Keywords:** esophageal squamous cell carcinoma, GLA, DNA damage repair, chemoresistance, migalastat

## Abstract

**Background:**

Esophageal squamous cell carcinoma (ESCC) is a highly lethal malignancy frequently complicated by chemoresistance. α-Galactosidase A (GLA), a lysosomal hydrolase associated with poor prognosis in multiple cancers, has not been investigated in ESCC.

**Methods:**

Two independent ESCC datasets (GSE161533, GSE38129) were used to screen commonly upregulated genes, which were ranked by diagnostic performance and further validated in an integrated single-cell RNA-seq atlas and an independent dataset (GSE20347), identifying GLA. GLA expression was validated by immunohistochemistry, qRT-PCR, and Western blot. Its biological function was assessed by siRNA-mediated knockdown combined with CCK-8, EdU, colony formation, Transwell, and wound healing assays. KEGG, GO, and GSEA were performed to explore underlying mechanisms. The effect of GLA knockdown on chemosensitivity, and the combination of the GLA pharmacological chaperone Migalastat with gemcitabine or cisplatin, were evaluated by CCK-8 assays in two ESCC cell lines.

**Results:**

GLA was significantly upregulated in ESCC, showed strong diagnostic performance across datasets, and was specifically enriched in malignant epithelial cells at the single-cell level. Multiple validation approaches confirmed GLA overexpression in ESCC cell lines and patient tissues. GLA knockdown markedly suppressed proliferation, colony formation, and migration. Enrichment analyses suggested that high GLA expression was associated with DNA damage repair, cell cycle, and DNA replication–related transcriptional programs, as well as with gemcitabine/cisplatin resistance-related genes. GLA knockdown increased the sensitivity of ESCC cells to gemcitabine and cisplatin, and Migalastat combined with either agent enhanced cytotoxicity in two ESCC cell lines, with combination index analysis indicating synergy.

**Conclusion:**

GLA is a novel, upregulated gene in ESCC with diagnostic relevance and an oncogenic role, and is associated with DNA damage repair–related transcriptional programs and chemotherapy response. Combining Migalastat with chemotherapy may represent a candidate strategy that warrants further mechanistic and *in vivo* investigation.

## Introduction

Esophageal cancer ranks as the seventh most commonly diagnosed cancer and the sixth leading cause of cancer-related mortality worldwide, with a 5-year survival rate of only approximately 22% (1, 2). The two predominant histological subtypes are esophageal squamous cell carcinoma (ESCC) and esophageal adenocarcinoma (EAC), which differ markedly in epidemiology and biology (3, 4). China bears more than half of the global esophageal cancer burden, with ESCC accounting for over 90% of cases (5, 6).

For unresectable or locally advanced ESCC, chemotherapy-based combination therapy remains the cornerstone of clinical management (7, 8). First-line regimens primarily include fluorouracil plus platinum (FP) or paclitaxel/docetaxel plus platinum (TP) (7). Nevertheless, intrinsic or acquired chemoresistance still accounts for a large proportion of treatment failures (9, 10), and there is an urgent need for novel therapeutic targets capable of sensitizing tumors to chemotherapy.

α-Galactosidase A (GLA) is a lysosomal hydrolase that catalyzes the hydrolysis of α-D-galactose residues from globotriaosylceramide (Gb3) (11).Loss-of-function mutations in GLA cause Fabry disease, an X-linked lysosomal storage disorder characterized by abnormal Gb3 accumulation (12, 13). Recent studies have reported that GLA is highly expressed and associated with poor prognosis in multiple cancers, including low-grade glioma (LGG), liver hepatocellular carcinoma (LIHC), stomach adenocarcinoma (STAD), adrenocortical carcinoma (ACC), uveal melanoma (UVM), and glioblastoma multiforme (GBM), suggesting that GLA may broadly contribute to tumor progression across cancer types (14). However, its expression, biological function, and role in chemoresistance in ESCC remain unexplored.

In this study, by combining differential expression screening across multiple independent ESCC datasets, diagnostic ranking, and single-cell analysis, we identified GLA as a significantly upregulated gene that is specifically enriched in malignant epithelial cells of ESCC, and validated its expression in patient tissues by immunohistochemistry. The oncogenic function of GLA was further confirmed through small interfering RNA (siRNA)-mediated knockdown and *in vitro* proliferation, migration, and colony formation assays, while Kyoto Encyclopedia of Genes and Genomes (KEGG), Gene Ontology (GO), and Gene Set Enrichment Analysis (GSEA) enrichment and drug sensitivity analyses were performed to elucidate its underlying mechanisms.

## Materials & methods

### Database mining and bioinformatic analyses

Three independent ESCC microarray datasets were retrieved from the Gene Expression Omnibus (GEO) database (https://www.ncbi.nlm.nih.gov/geo/): GSE161533 (28 paired tumor–normal samples) and GSE38129 (30 paired tumor–normal samples) were used for candidate gene screening, and GSE20347 (17 paired tumor–normal samples) was used for independent external validation. Probe-level data were annotated to gene symbols, and differential expression analysis was performed for each dataset using the *limma* R package (version 3.66.0), with the thresholds set as |log_2_FC| > 0.585 (fold change > 1.5) and adjusted *P* < 0.05. Functional enrichment analyses, including Gene Ontology (GO), Kyoto Encyclopedia of Genes and Genomes (KEGG) pathway, and Gene Set Enrichment Analysis (GSEA), were conducted using the *clusterProfiler* R package. All analyses were performed in R software (version 4.5.2). To prioritize candidate genes, DEGs that were consistently upregulated in both the GSE161533 and GSE38129 screening datasets were intersected. The diagnostic performance of each commonly upregulated gene was then evaluated in GSE161533 by receiver operating characteristic (ROC) analysis using the *pROC* package, and genes were ranked by the area under the curve (AUC); the top 10 genes were retained for single-cell validation.

### Single-cell RNA-seq analysis

An integrated single-cell RNA-seq (scRNA-seq) atlas of ESCC was constructed from five publicly available datasets (GSE145370, GSE160269, GSE188900, GSE196756, and GSE203115), comprising a total of 174,946 cells. Data were processed and integrated using the *Seurat* R package with batch effects corrected by *Harmony*. Cells were clustered and visualized by uniform manifold approximation and projection (UMAP) and annotated into major cell types using canonical markers. Within the epithelial compartment, malignant and non-malignant epithelial cells were distinguished on the basis of established markers (e.g., EPCAM, KRT5, KRT14, TP63, SOX2, and PCNA for malignant cells; KRT4, KRT13, and CRNN for normal differentiated epithelium). Differences in candidate gene expression between malignant and non-malignant epithelial cells were assessed using the Wilcoxon rank-sum test, with *P* values adjusted by the Benjamini–Hochberg method.

### Clinical samples and immunohistochemistry

Paired tumor and adjacent non-tumor tissues were collected from 5 patients with histologically confirmed ESCC. All participants provided written informed consent prior to enrollment, and the study protocol was approved by the Ethics Committee of the Fourth Affiliated Hospital of Soochow University. Immunohistochemistry was performed on paraffin-embedded sections. Briefly, sections were deparaffinized in xylene and rehydrated through a graded ethanol series. Heat-induced antigen retrieval was carried out in sodium citrate buffer at 95°C, followed by endogenous peroxidase blocking. The sections were then incubated overnight at 4°C with anti-GLA primary antibody (Proteintech, rabbit IgG, 1:1000). On the following day, sections were incubated with the corresponding secondary antibody for 2 h at room temperature, followed by 3,3’-diaminobenzidine (DAB) chromogenic development and hematoxylin counterstaining. Finally, the sections were mounted and examined under a light microscope. For semi-quantitative analysis, three representative fields per section were captured, and the GLA-positive area was quantified using ImageJ by two investigators blinded to the group assignment; the average value was used for statistical comparison between tumor and adjacent normal tissues.

### Cell lines and cell culture

The human normal esophageal epithelial cell line Het-1A and four esophageal squamous carcinoma cell lines (KYSE-30, KYSE-150, KYSE-180, and TE-1) were obtained from EallBio Technologies (Beijing, China). All cell lines were authenticated by short tandem repeat (STR) profiling and confirmed to be free of mycoplasma contamination. KYSE-30, KYSE-150, KYSE-180, and TE-1 cells were cultured in RPMI-1640 medium (Gibco, New York, USA) supplemented with 10% fetal bovine serum (Gibco) and 1% penicillin–streptomycin (Beyotime, Shanghai, China). Het-1A cells were maintained in DMEM medium (Gibco) supplemented with the same additives. All cell lines were incubated at 37°C in a humidified atmosphere containing 5% CO_2_.

### Cell transfection

All plasmids were synthesized by Gemma Gene (Shanghai, China), and the siRNAs were synthesized by Qingke (Beijing, China). Cell transfection was conducted using Lipofectamine 3000 (Thermo Fisher Scientific, California, USA) in accordance with the manufacturer’s instructions. The siRNA sequences used in this study are provided in **Supplementary Table 1**.

### RNA extraction and quantitative real-time PCR

Total RNA was extracted from cells or tissues using TRIzol reagent, followed by reverse transcription into cDNA using a reverse transcription kit (Vazyme, Nanjing, China). Quantitative real-time PCR (qRT-PCR) was performed using SYBR Green Mix (Vazyme, Nanjing, China). Relative RNA expression levels were calculated using the 2^-ΔΔCt^ method and normalized to the internal reference gene GAPDH. The primer sequences used in this study are listed in **Supplementary Table 1**.

### Cell proliferation assay

CCK-8 Assay: ESCC cells were seeded into 96-well plates at a density of 2×10^3^ cells per well. At 0, 24, 48, 72, and 96 h, 10 μL of CCK-8 reagent (Vazyme, Nanjing, China) was added to each well. After incubation for 2 h, the absorbance at 450 nm was measured using a microplate reader (BioTek Synergy H1).

Colony Formation Assay:A total of 1×10^3^ ESCC cells were seeded into each well of a 6-well plate. During the culture period, the medium was replaced every 4 days. After 2 weeks, the formed colonies were fixed with 4% paraformaldehyde for 15 min, stained with 0.1% crystal violet solution for 20 min, and subsequently washed with phosphate-buffered saline (PBS). Colony numbers were quantified using ImageJ software.

EdU Assay: ESCC cells were seeded into 24-well plates and cultured overnight. Cell proliferation was evaluated using an EdU Cell Proliferation Kit (Beyotime, Shanghai, China) according to the manufacturer’s instructions. Briefly, cells were incubated with EdU working solution for 2 h at 37°C, followed by fixation with 4% paraformaldehyde for 15 min and permeabilization with 0.3% Triton X-100 for 15 min. Subsequently, cells were incubated with Click Reaction Solution in the dark for 30 min and counterstained with DAPI to visualize nuclei. Images were captured using a fluorescence microscope, and the percentage of EdU-positive cells was quantified using ImageJ software.

### Cell migration assays

Transwell Assay: A total of 4 × 10^4^ serum-starved ESCC cells were resuspended in 200 μL of serum-free medium and seeded into the upper chamber of a Transwell insert (Corning, NY, USA), while 700 μL of complete medium was added to the lower chamber. After 36 h of incubation, cells that had migrated through the membrane were fixed with 4% paraformaldehyde for 15 min and stained with 0.5% crystal violet solution (Beyotime, Shanghai, China) for 20 min. Non-migrated cells remaining on the upper surface of the membrane were gently removed using a cotton swab. Images from three randomly selected fields were captured under an inverted microscope. Quantitative analysis of the images was performed using ImageJ software.

Wound Healing Assay: ESCC cells were cultured in 6-well plates until approximately 90% confluence was reached. A linear wound was generated in the cell monolayer using a 200 μL pipette tip. The cells were then washed with PBS to remove detached cells and debris, followed by incubation in serum-free medium. Images of the wound area were captured at 0 h and 24 h using an inverted microscope to assess cell migratory ability.

### Western blot

Cells or tissues were lysed in 100 μL of protein lysis buffer (Punoon, Nanjing, China; RIPA lysis buffer supplemented with protease inhibitor at a ratio of 100:1). Total proteins were separated by SDS-PAGE and subsequently transferred onto PVDF membranes (Millipore, Massachusetts, USA). After blocking with 5% skim milk in TBST for 2 h, the membranes were incubated with primary antibodies at 4°C for at least 12 h. The primary antibodies and their dilution ratios were as follows: GLA (1:1000) and Vinculin (1:1000). All primary antibodies were purchased from Abclonal (Wuhan, China). The membranes were then incubated with the corresponding secondary antibodies (1:10,000; Thermo Fisher, California, USA) for 2 h at room temperature. Protein bands were visualized using an enhanced chemiluminescence (ECL) substrate (Vazyme, Nanjing, China) and detected with an imaging analysis system (Tanon, Shanghai, China).

### Drug combination assays

Migalastat (Cat. # HY-14929), gemcitabine (Cat. # HY-17026), and cisplatin (Cat. # HY-17394) were all purchased from MedChemExpress (MCE). Migalastat was used at 0.04 μM, the reported IC50 against human α-galactosidase A provided by the manufacturer (MedChemExpress) (15). The working concentrations of gemcitabine and cisplatin were determined based on their respective half maximal inhibitory concentrations (IC50) in KYSE-150 and TE-1 cells. To assess the effect of GLA depletion on chemosensitivity, KYSE-150 and TE-1 cells transfected with si-NC or si-GLA were treated with a concentration gradient of gemcitabine or cisplatin, and the half-maximal inhibitory concentration (IC_50_) was determined by CCK-8 assay. To evaluate the interaction between Migalastat and chemotherapy, a 6×6 dose-matrix combination assay was performed in KYSE-150 and TE-1 cells, and the combination index (CI) was calculated using CompuSyn software, with CI < 1 indicating synergy.

### Statistical analysis

Statistical analyses and graph generation were performed using GraphPad Prism 9. Bioinformatics analyses were conducted using R (version 4.5.2) and RStudio (https://posit.co/download/rstudio-desktop/). Two-group comparisons of gene expression were performed using the Wilcoxon rank-sum test or unpaired/paired Student’s t-test (as appropriate). Diagnostic performance was evaluated by ROC analysis and quantified by the AUC. Spearman correlation analysis was used to assess associations between continuous variables. IC50 values were calculated by nonlinear regression of dose-response curves in GraphPad Prism 9. All *in vitro* experiments were repeated at least three times. Data are expressed as mean ± standard deviation (SD). A two-tailed p-value < 0.05 was considered statistically significant.

## Results

### Identification of GLA as a candidate upregulated gene in ESCC through integrated bulk and single-cell analysis

**Figure 1:**
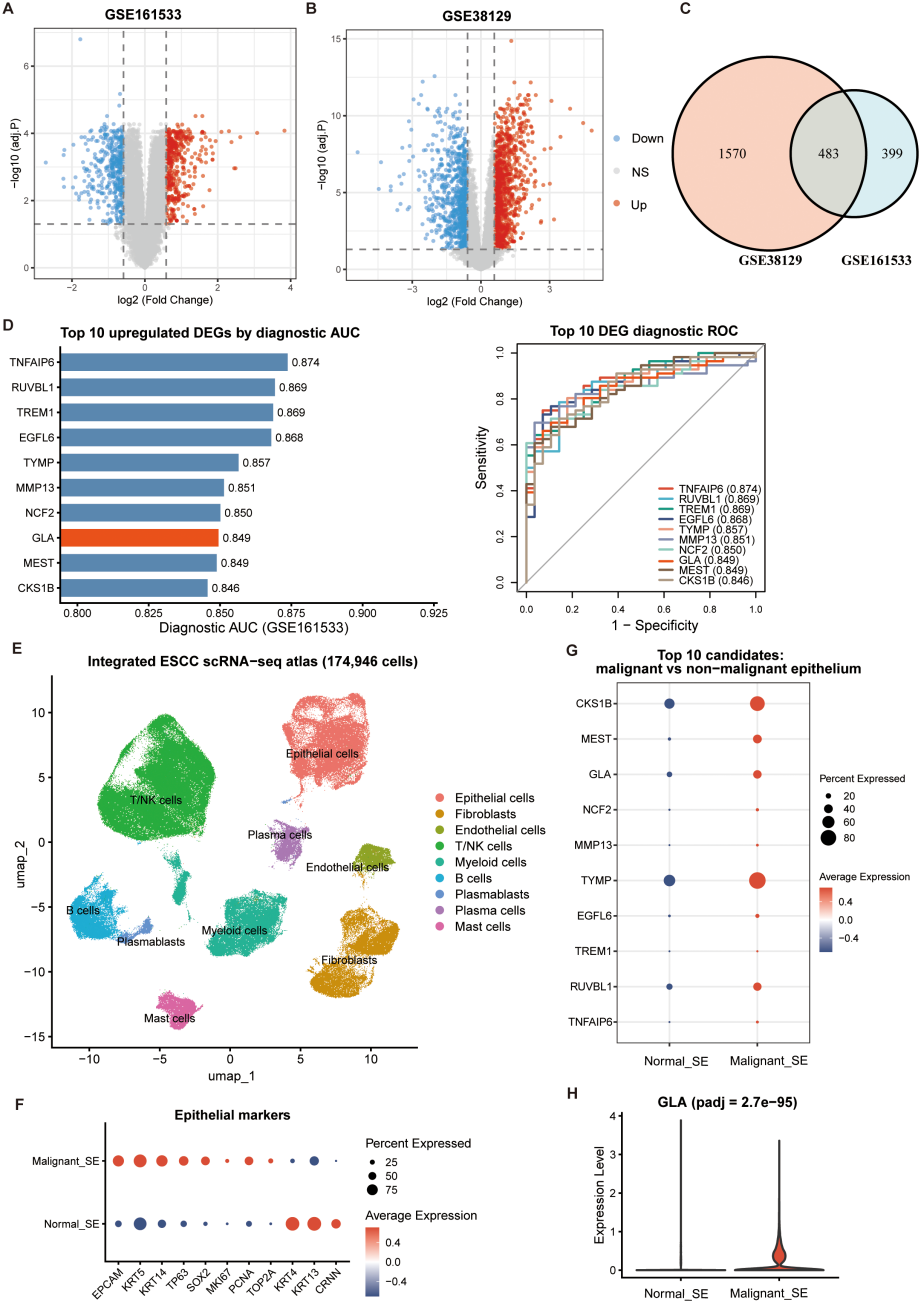
Identification of GLA as an upregulated candidate gene in ESCC through integrated bulk and single-cell analysis. **(A, B)** Volcano plots of differentially expressed genes (DEGs) between tumor and paired adjacent normal tissues in GSE161533 **(A)** and GSE38129 **(B)**; red, upregulated; blue, downregulated; grey, not significant (|log_2_FC| > 0.585, adjusted *P* < 0.05). **(C)** Venn diagram of DEGs from the two datasets, showing 483 common DEGs. **(D)** Commonly upregulated DEGs ranked by diagnostic performance (ROC AUC) in GSE161533; the top 10 genes are shown, with GLA highlighted (left), and their corresponding ROC curves (right). **(E)** UMAP of an integrated ESCC single-cell RNA-seq atlas comprising 174,946 cells from five public datasets, annotated into nine major cell types. **(F)** Dot plot of canonical markers distinguishing malignant (EPCAM, KRT5, KRT14, TP63, SOX2, PCNA) from non-malignant (KRT4, KRT13, CRNN) epithelial cells. **(G)** Dot plot of the top 10 candidate genes in malignant versus non-malignant epithelial cells. **(H)** Violin plot of GLA expression in malignant versus non-malignant epithelial cells (Wilcoxon, adjusted *P = 2.7 × 10^-95^*).

To identify candidate genes specifically dysregulated in ESCC, we analyzed two independent ESCC microarray datasets. Differential expression analysis between tumor and paired adjacent normal tissues identified DEGs in GSE161533 (**Figure 1A**) and GSE38129 (**Figure 1B**), and intersection of the two datasets yielded 483 common DEGs (**Figure 1C**). To prioritize candidates with diagnostic relevance, commonly upregulated DEGs were ranked by their diagnostic performance (ROC AUC) in GSE161533, and the top 10 genes were retained for further validation (**Figure 1D**). To examine the cellular origin of these candidates, we constructed an integrated ESCC single-cell RNA-seq atlas comprising 174,946 cells from five public datasets, which were annotated into nine major cell types (**Figure 1E**). Malignant and non-malignant epithelial cells were distinguished using canonical markers, with malignant cells showing higher expression of EPCAM, KRT5, KRT14, TP63, SOX2, and PCNA, and non-malignant epithelium expressing the differentiation markers KRT4, KRT13, and CRNN (**Figure 1F**). Among the top 10 candidates, most were proliferation- or stroma-associated genes already characterized in ESCC, whereas GLA, a lysosomal hydrolase whose expression and function in ESCC remain unexplored, was significantly enriched in malignant epithelial cells (**Figure 1G**). GLA expression was markedly higher in malignant than in non-malignant epithelial cells (Wilcoxon, adjusted P = 2.7 × 10^−95^; **Figure 1H**). Notably, most of the other top-ranked candidates—such as TNFAIP6, RUVBL1, TREM1, MMP13, and CKS1B—have already been functionally characterized in ESCC as proliferation-, invasion-, or inflammation-related genes. In contrast, GLA is a lysosomal hydrolase that has not been studied in ESCC and is pharmacologically targetable by the FDA-approved agent Migalastat. Given its consistent upregulation, malignant-epithelium-specific expression, biological novelty, and translational tractability, GLA was selected for in-depth investigation.

### GLA is highly expressed in both ESCC cell lines and patient samples

**Figure 2:**
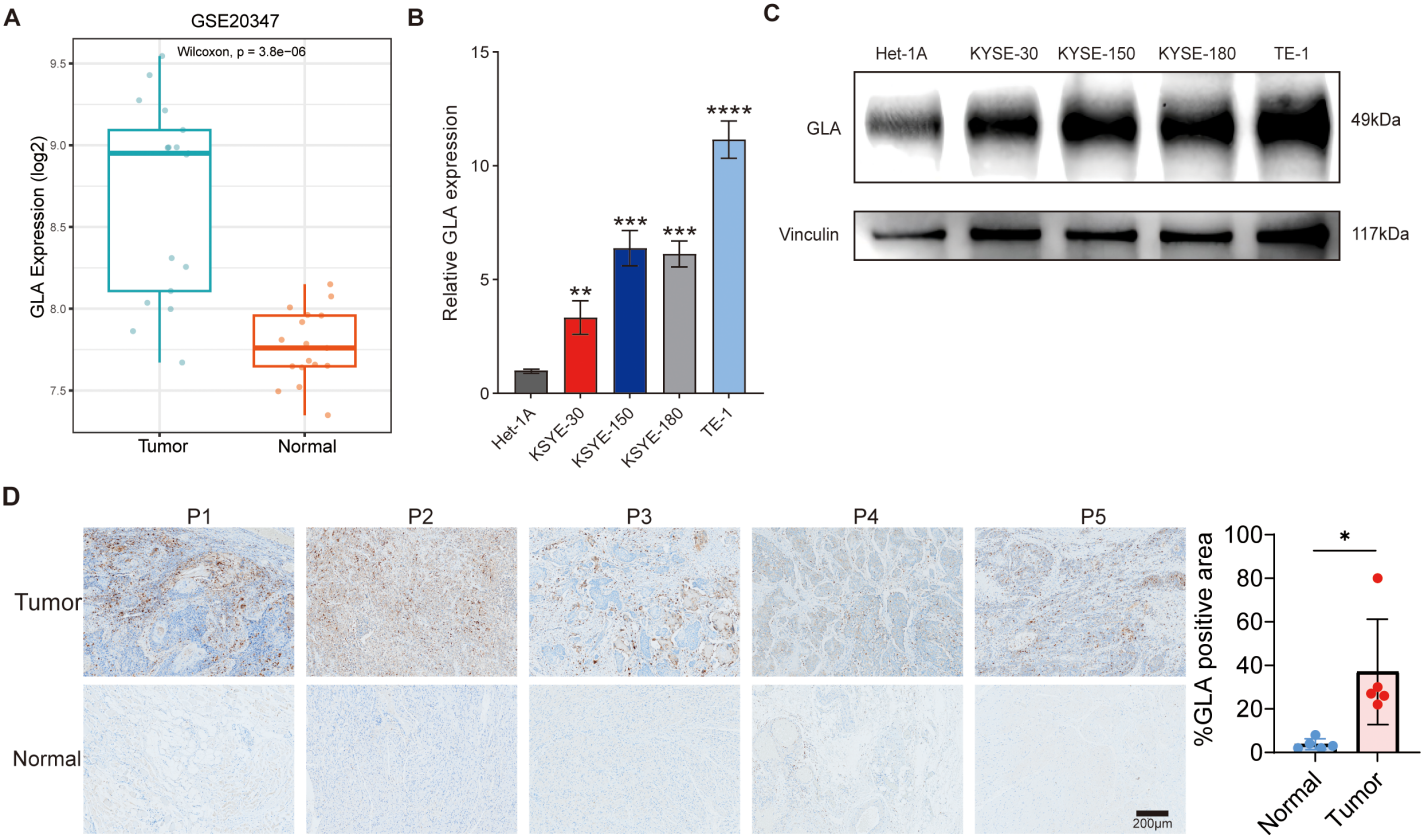
Validation of GLA upregulation in ESCC cell lines and patient tissues. **(A)** External validation of GLA expression in the independent GSE20347 cohort (17 paired tumor–normal samples; Wilcoxon, *P* = 3.8 × 10^-6^). **(B, C)** qRT-PCR **(B)** and Western blot **(C)** analysis of GLA expression in four ESCC cell lines (KYSE-30, KYSE-150, KYSE-180, TE-1) relative to the normal esophageal epithelial cell line Het-1A; Vinculin served as loading control. **(D)** Representative immunohistochemical staining of GLA in tumor and paired adjacent normal tissues from five ESCC patients (P1–P5; left), with quantification of GLA-positive area (right). Scale bar: 200 μm. *P < 0.05, **P < 0.01, ***P < 0.001, ****P < 0.0001..

To further validate the expression of GLA in ESCC, we performed external validation using the GSE20347 dataset, which showed that GLA was significantly upregulated in tumor tissues compared with adjacent non-tumor tissues (**Figure 2A**). Consistently, qRT-PCR (**Figure 2B**) and Western blot (**Figure 2C**) analyses revealed that, compared with the normal human esophageal epithelial cell line Het-1A, GLA expression was markedly elevated in multiple ESCC cell lines, including KYSE-30, KYSE-150, KYSE-180, and TE-1. Given that KYSE-150 and TE-1 displayed the highest GLA expression levels among the cell lines tested, these two cell lines were selected for subsequent functional experiments. Furthermore, immunohistochemistry (IHC) staining of tumor and paired adjacent non-tumor tissues from 5 ESCC patients showed stronger GLA staining in tumor tissues, and blinded semi-quantitative analysis confirmed a significantly higher GLA-positive area in tumor than in paired adjacent normal tissues (**Figure 2D**). Taken together, these findings demonstrated that GLA was markedly upregulated in ESCC at both the transcriptional and protein levels.

### Knockdown of GLA significantly suppressed the proliferation, colony formation, and migration of ESCC cells

**Figure 3:**
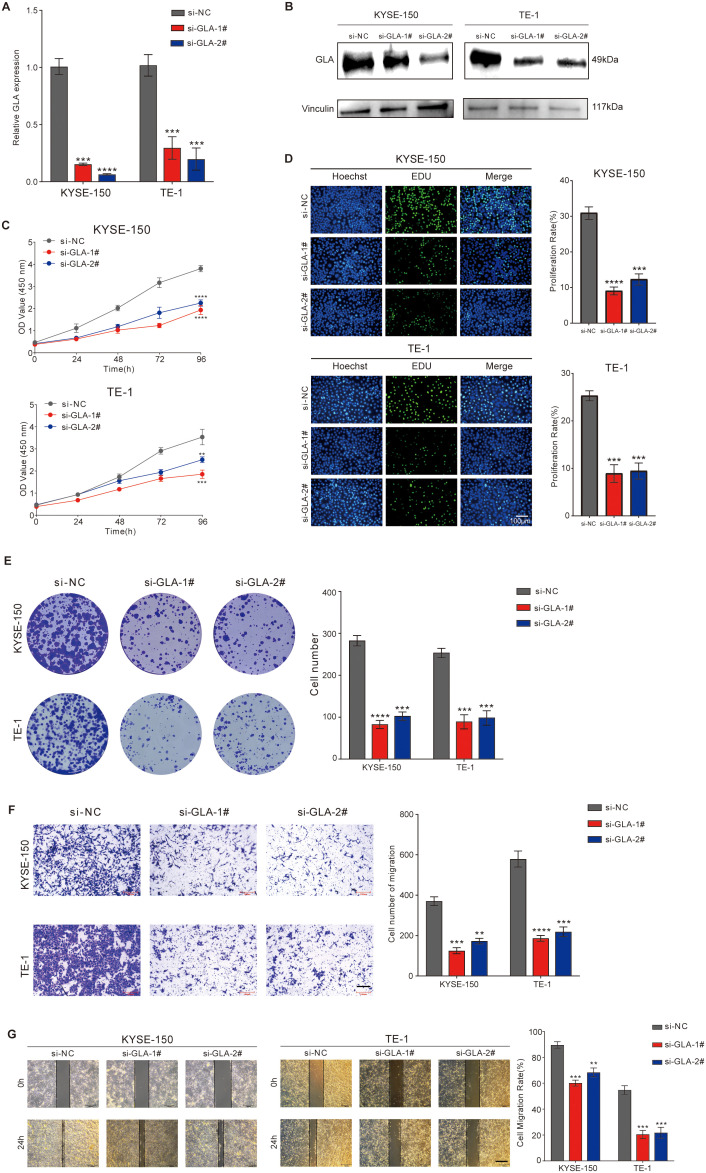
Knockdown of GLA expression inhibited the proliferation, colony formation, and migration of ESCC cells. **(A)** qRT-PCR validation of GLA knockdown efficiency in KYSE-150 and TE-1 cells transfected with si-NC, si-GLA-1#, or si-GLA-2#. **(B)** Western blot validation of GLA knockdown. **(C)** CCK-8 assay to detect changes in cell viability within 96 h after GLA knockdown. **(D)** EdU incorporation assay showing decreased proliferation rate (representative images and quantification). Scale bar: *100 μm.* **(E)** Colony formation assay. **(F)** Transwell migration assay. Scale bar: *100 μm.* **(G)** Wound healing assay at 0 h and 24 h. *P<0.05, **P<0.01, ***P<0.001, ****P<0.0001..

To investigate the biological function of GLA, we designed two specific small interfering RNAs targeting GLA (si-GLA-1# and si-GLA-2#), and their knockdown efficiency was confirmed in both KYSE-150 and TE-1 cells by qRT-PCR and Western blot (**Figures 3A, B**). CCK-8 assay showed that downregulation of GLA significantly inhibited the proliferative capacity of KYSE-150 and TE-1 cells (**Figure 3C**). EdU incorporation assay further demonstrated that, compared with the control group, the proportion of EdU-positive cells was markedly decreased in ESCC cells after GLA knockdown (**Figure 3D**). Colony formation assay further confirmed that silencing GLA substantially reduced the number of colonies formed by both ESCC cell lines (**Figure 3E**). In addition, Transwell assay revealed that the number of migrated cells in the si-GLA-1# and si-GLA-2# groups was significantly lower than that in the si-NC group (**Figure 3F**). Wound healing assay similarly showed that inhibition of GLA markedly impaired the wound closure ability of KYSE-150 and TE-1 cells (**Figure 3G**). Collectively, these results indicated that GLA may promote the proliferation and migration of ESCC cells.

### GLA expression correlates with DNA damage repair–related transcriptional programs and chemotherapy response

**Figure 4:**
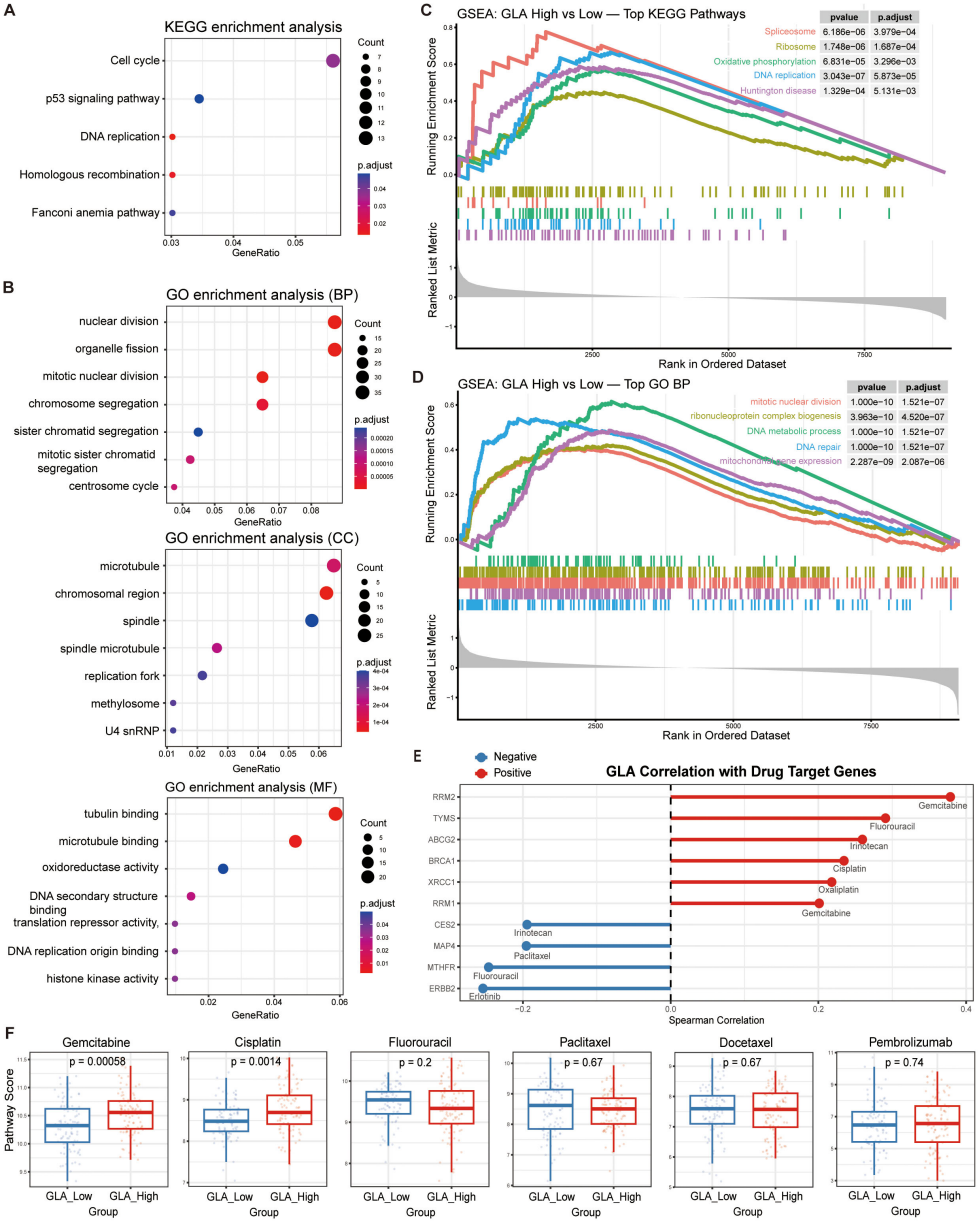
GLA is associated with DNA damage repair pathways and chemotherapy resistance in ESCC. **(A, B)** KEGG pathway **(A)** and GO **(B)** enrichment analysis of DEGs between GLA-high and GLA-low groups (BP, biological process; CC, cellular component; MF, molecular function). **(C)** GSEA plot showing the most enriched KEGG pathway in the GLA-high group. **(D)** GSEA plot showing the most enriched GO biological process in the GLA-high group. **(E)** Lollipop plot of correlations between GLA expression and chemoresistance-related drug-target genes. **(F)** Box plots comparing drug-response pathway activity scores for six agents (gemcitabine, cisplatin, fluorouracil, pembrolizumab, paclitaxel, and docetaxel) between GLA-high and GLA-low groups. **(G)** CCK-8 assay assessing the sensitivity of ESCC cells to gemcitabine or cisplatin following GLA knockdown (si-NC vs. si-GLA). **(H)** CCK-8 assay evaluating the combination of the GLA-targeting pharmacological chaperone Migalastat with gemcitabine or cisplatin in ESCC cells. **(I)** CCK-8 dose-matrix assay of Migalastat combined with gemcitabine or cisplatin, analyzed for synergy. *P < 0.05, **P < 0.01..

To explore biological pathways associated with GLA expression in ESCC, we performed functional enrichment analyses on the differentially expressed genes between the GLA-high and GLA-low groups. KEGG enrichment analysis (**Figure 4A**) revealed that the enriched pathways were mainly involved in cell cycle, p53 signaling pathway, DNA replication, and homologous recombination. GO analysis (**Figure 4B**) showed that, at the biological process (BP) level, the genes were predominantly enriched in nuclear division, chromosome segregation, and mitotic processes; at the cellular component (CC) level, in microtubule, chromosomal region, and replication fork structures; and at the molecular function (MF) level, in microtubule binding and DNA replication origin binding activities. GSEA further confirmed that high GLA expression was significantly positively correlated with the spliceosome, ribosome, oxidative phosphorylation, and DNA replication pathways at the KEGG level, and with mitotic nuclear division, DNA metabolic process, and DNA repair at the GO biological-process level (**Figures 4C, D**).

To evaluate the influence of GLA expression on chemotherapeutic sensitivity, we analyzed the correlation between GLA and chemotherapy-related drug target genes. The results showed that GLA was significantly positively correlated with several chemoresistance-related genes, including RRM2 and RRM1 (associated with gemcitabine resistance), TYMS (the target of 5-FU), BRCA1 and XRCC1 (associated with platinum resistance), and the multidrug resistance efflux pump ABCG2 (**Figure 4E**). Drug pathway activity analysis further revealed that the resistance pathways for gemcitabine (*P*=0.00058) and cisplatin (*P*=0.0014) were significantly activated in the GLA-high group, whereas no significant differences were observed for 5-FU, paclitaxel, docetaxel, or pembrolizumab (**Figure 4F**).

**Figure 5:**
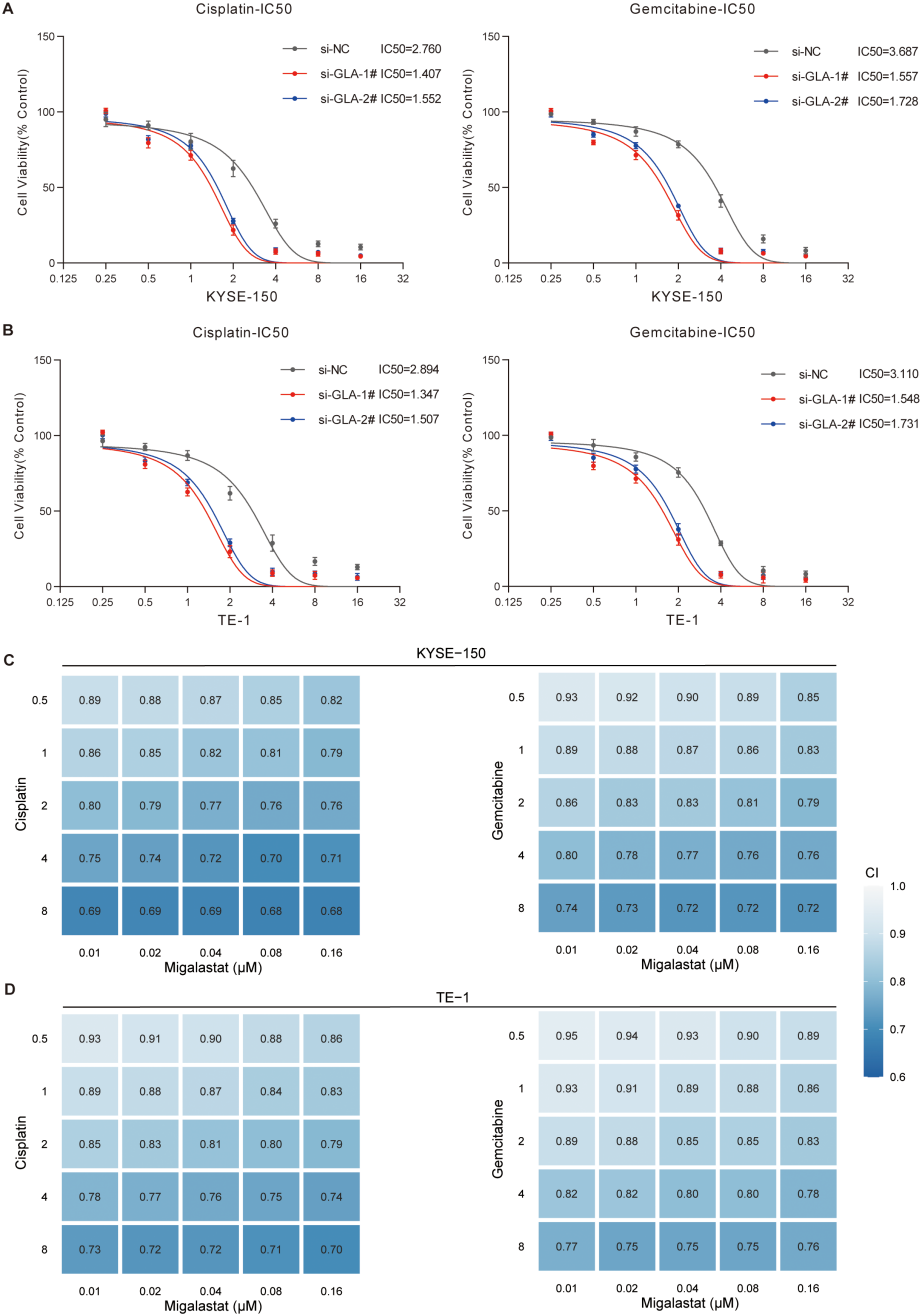
GLA knockdown sensitizes ESCC cells to chemotherapy, and Migalastat synergizes with chemotherapeutic agents. **(A, B)** CCK-8 dose–response curves and IC50 values for cisplatin and gemcitabine in KYSE-150 **(A)** and TE-1 **(B)** cells transfected with si-NC, si-GLA-1#, or si-GLA-2#. GLA knockdown markedly reduced the IC50 of both agents. **(C, D)** Combination index (CI) heatmaps for Migalastat combined with cisplatin or gemcitabine in KYSE-150 **(C)** and TE-1 **(D)** cells across a dose matrix. All tested combinations showed CI < 1 (range 0.68–0.95), indicating synergy.

To directly test whether GLA influences chemosensitivity, we compared the response of si-NC and si-GLA cells to gemcitabine and cisplatin. GLA knockdown lowered the IC50 of both gemcitabine and cisplatin in KYSE-150 and TE-1 cells, indicating that depletion of GLA sensitized ESCC cells to chemotherapy (**Figures 5A, B**). To further explore the feasibility of pharmacologically targeting GLA, Migalastat was combined with gemcitabine or cisplatin in both cell lines using a dose-matrix design, and the interaction was evaluated by Chou-Talalay analysis. Across all tested dose combinations, the combination index (CI) was < 1 (range 0.68–0.95) for Migalastat combined with either gemcitabine or cisplatin, indicating a consistent synergistic effect (**Figures 5C, D**). These results suggested that both genetic depletion and pharmacological targeting of GLA enhance the sensitivity of ESCC cells to gemcitabine and cisplatin, consistent with the possibility that GLA may influence chemotherapy response through biological programs related to DNA repair and nucleotide metabolism, and indicating the potential translational value of this combination strategy.

## Discussion

In this study, we identified GLA as a significantly upregulated gene in ESCC and explored its potential tumor-promoting role through multiple experimental approaches. Functional enrichment analyses suggested that high GLA expression is associated with DNA damage repair–related transcriptional programs, which may in turn contribute to chemoresistance. While previous studies have reported GLA as a poor prognostic gene associated with immune infiltration in low-grade glioma and hepatocellular carcinoma (14), our work is the first to suggest a potential tumor-promoting role for GLA in ESCC and its possible involvement in the DNA repair–chemoresistance axis.

The mechanistic basis for the association between GLA and chemoresistance is not yet fully defined. As a lysosomal hydrolase, GLA participates in glycosphingolipid catabolism, and altered GLA expression may in principle contribute to broader reprogramming of cellular lipid metabolism (16, 17). Such lipid remodeling can activate oncogenic signaling pathways including PI3K/AKT and MAPK (18, 19), both of which can influence tumor proliferation and survival. However, glycosphingolipid metabolism in tumors is highly dynamic and its net effect is context-dependent, and we did not directly measure Gb3 levels or GLA enzymatic activity in this study. Rather than this lipid-centered route, our transcriptomic data more consistently linked high GLA expression to DNA damage repair and nucleotide-metabolism programs (e.g., RRM2, which was upregulated across all three independent cohorts, together with BRCA1 and TYMS), which we therefore regard as the more likely axis connecting GLA to chemotherapy response in ESCC (20, 21). These mechanisms together offer a biological context for our observation that high GLA expression is associated with DNA damage repair–related transcriptional programs and with reduced sensitivity to gemcitabine and cisplatin in ESCC.

Migalastat is a small-molecule ligand of α-galactosidase A that was originally approved by the FDA for Fabry disease, with a well-characterized safety and pharmacokinetic profile in clinical use (22–24). Its utility in oncology, however, remains largely unexplored. Repositioning Migalastat for cancer treatment fits the framework of “old drug, new use”, an approach that takes advantage of established safety data to shorten development timelines and reduce the cost of clinical translation (25, 26). Our data show that Migalastat, when combined with gemcitabine or cisplatin, markedly enhanced cytotoxicity against ESCC cells, offering an early but encouraging basis for further translational studies in ESCC. Under the continuous exposure conditions used in this study, Migalastat phenocopied the chemosensitizing effect observed following GLA knockdown; however, the extent to which this effect is mediated through direct modulation of GLA activity remains to be determined.

Several limitations of this study should be noted. First, *in vivo* validation in mouse models is still needed to further support our findings. Second, the clinical IHC analysis was based on only 5 paired samples, and larger cohorts are required for confirmation. Third, the proposed involvement of GLA in the DNA damage repair pathway was inferred primarily from KEGG, GO, and GSEA enrichment analyses of GLA-high versus GLA-low transcriptomes. These analyses are hypothesis-generating and do not establish that GLA directly regulates DNA damage repair. Direct functional validation—such as γ-H2AX and RAD51 foci formation, comet assays, and protein-level assessment of key repair factors following GLA perturbation—was beyond the scope and timeframe of the present study and represents an important direction for future work. Finally, the precise mechanism by which Migalastat modulates GLA activity in tumors remains to be determined and requires more dedicated experimental investigation, including direct measurement of GLA enzymatic activity. In addition, our functional data were based on siRNA-mediated knockdown; rescue and gain-of-function (overexpression) experiments were not performed and will be needed to fully confirm the specificity and sufficiency of GLA. Because GLA knockdown also reduced cell proliferation, the observed effects in the Transwell and wound-healing assays may be partly influenced by decreased proliferation; proliferation-controlled migration assays are warranted in future studies.

In future work, we plan to assemble an in-house RNA-seq cohort of ESCC patients and combine it with single-cell sequencing to characterize the expression and distribution of GLA across different tumor cell subpopulations. Patient-derived organoid models will also be established to validate the proposed combination therapy strategy. Further studies will also focus on dissecting the precise molecular mechanism by which GLA is associated with DNA damage repair to build a more comprehensive mechanistic basis for targeting GLA in ESCC.

## Conclusion

In summary, this study identified GLA as a significantly upregulated gene in esophageal squamous cell carcinoma (ESCC) that is specifically enriched in malignant epithelial cells and shows strong diagnostic performance across multiple independent cohorts. Through clinical samples and functional experiments, we validated the high expression of GLA in ESCC tissues and cells, and confirmed that GLA knockdown significantly inhibited the malignant progression of ESCC. Enrichment analysis further revealed that high GLA expression is associated with DNA damage repair–related transcriptional programs and may contribute to chemotherapy resistance.

## Data Availability

All datasets analyzed in this study are publicly available. The microarray datasets GSE161533, GSE38129, and GSE20347 were obtained from the Gene Expression Omnibus (https://www.ncbi.nlm.nih.gov/geo/), and the single-cell RNA-seq datasets (GSE145370, GSE160269, GSE188900, GSE196756, and GSE203115) were obtained from the same repository. Any additional data are available from the corresponding author upon reasonable request. The data that support the findings of this study are available from the corresponding author upon reasonable request.

## Supplementary material

Supplementary Table 1 Experimental primer sequences. Name Sequence (5’-3’)

## References

[1] Bray F Laversanne M Sung H Ferlay J Siegel RL Soerjomataram I . Global cancer statistics 2022: GLOBOCAN estimates of incidence and mortality worldwide for 36 cancers in 185 countries. CA Cancer J Clin. (2024) 74:229–63. doi: 10.3322/caac.21834 38572751

[2] Siegel RL Kratzer TB Giaquinto AN Sung H Jemal A . Cancer statistics, 2025. CA Cancer J Clin. (2025) 75:10–45. doi: 10.3322/caac.21871 39817679 PMC11745215

[3] Smyth EC Lagergren J Fitzgerald RC Lordick F Shah MA Lagergren P . Oesophageal cancer. Nat Rev Dis Primers. (2017) 3:17048. doi: 10.1016/s0140-6736(17)31462-9 28748917 PMC6168059

[4] Arnold M Soerjomataram I Ferlay J Forman D . Global incidence of oesophageal cancer by histological subtype in 2012. Gut. (2015) 64:381–7. doi: 10.1136/gutjnl-2014-308124 25320104

[5] Han B Zheng R Zeng H Wang S Sun K Chen R . Cancer incidence and mortality in China, 2022. J Natl Cancer Cent. (2024) 4:47–53. doi: 10.1016/j.jncc.2024.01.006 39036382 PMC11256708

[6] Chen W Zheng R Baade PD Zhang S Zeng H Bray F . Cancer statistics in China, 2015. CA Cancer J Clin. (2016) 66:115–32. doi: 10.3322/caac.21338 26808342

[7] Ajani JA D'Amico TA Bentrem DJ Cooke D Corvera C Das P . Esophageal and esophagogastric junction cancers, version 2.2023, NCCN clinical practice guidelines in oncology. J Natl Compr Canc Netw. (2023) 21:393–422. doi: 10.6004/jnccn.2023.0019 37015332

[8] van Hagen P Hulshof MC van Lanschot JJ Steyerberg EW van Berge Henegouwen MI Wijnhoven BP . Preoperative chemoradiotherapy for esophageal or junctional cancer. N Engl J Med. (2012) 366:2074–84. doi: 10.1056/nejmoa1112088 22646630

[9] Vasan N Baselga J Hyman DM . A view on drug resistance in cancer. Nature. (2019) 575:299–309. doi: 10.1038/s41586-019-1730-1 31723286 PMC8008476

[10] Holohan C Van Schaeybroeck S Longley DB Johnston PG . Cancer drug resistance: an evolving paradigm. Nat Rev Cancer. (2013) 13:714–26. doi: 10.1038/nrc3599 24060863

[11] Garman SC Garboczi DN . The molecular defect leading to Fabry disease: structure of human alpha-galactosidase. J Mol Biol. (2004) 337:319–35. doi: 10.2210/pdb1r46/pdb 15003450

[12] Germain DP . Fabry disease. Orphanet J Rare Dis. (2010) 5:30. doi: 10.1007/978-3-030-87893-1_46 21092187 PMC3009617

[13] Schiffmann R . Fabry disease. Pharmacol Ther. (2009) 122:65–77. doi: 10.1016/j.pharmthera.2009.01.003 19318041

[14] Zhang Y Li J Yin X . High-expression of Galactosidase alpha is correlated with poor prognosis and immune infiltration in low-grade glioma. J Cancer. (2023) 14:646–56. doi: 10.7150/jca.81975 37057282 PMC10088540

[15] Asano N Ishii S Kizu H Ikeda K Yasuda K Kato A . *In vitro* inhibition and intracellular enhancement of lysosomal alpha-galactosidase A activity in Fabry lymphoblasts by 1-deoxygalactonojirimycin and its derivatives. Eur J Biochem. (2000) 267:4179–86. doi: 10.1046/j.1432-1327.2000.01457.x 10866822

[16] Hannun YA Obeid LM . Sphingolipids and their metabolism in physiology and disease. Nat Rev Mol Cell Biol. (2018) 19:175–91. doi: 10.1038/nrm.2017.107 29165427 PMC5902181

[17] Russo D Capolupo L Loomba JS Sticco L D'Angelo G . Glycosphingolipid metabolism in cell fate specification. J Cell Sci. (2018) 131(24):jcs219204. doi: 10.1242/jcs.219204 30559216

[18] Mollinedo F Gajate C . Lipid rafts as signaling hubs in cancer cell survival/death and invasion: implications in tumor progression and therapy: Thematic Review Series: Biology of Lipid Rafts. J Lipid Res. (2020) 61:611–35. doi: 10.1194/jlr.tr119000439 33715811 PMC7193951

[19] Beloribi-Djefaflia S Vasseur S Guillaumond F . Lipid metabolic reprogramming in cancer cells. Oncogenesis. (2016) 5:e189. doi: 10.1038/oncsis.2015.49 26807644 PMC4728678

[20] Hinrichs JW Klappe K Hummel I Kok JW . ATP-binding cassette transporters are enriched in non-caveolar detergent-insoluble glycosphingolipid-enriched membrane domains (DIGs) in human multidrug-resistant cancer cells. J Biol Chem. (2004) 279:5734–8. doi: 10.1074/jbc.m306857200 14627714

[21] Greenlee JD Subramanian T Liu K King MR . Rafting down the metastatic cascade: the role of lipid rafts in cancer metastasis, cell death, and clinical outcomes. Cancer Res. (2021) 81:5–17. doi: 10.1158/0008-5472.can-20-2199 32999001 PMC7952000

[22] Germain DP Hughes DA Nicholls K Bichet DG Giugliani R Wilcox WR . Treatment of fabry's disease with the pharmacologic chaperone migalastat. N Engl J Med. (2016) 375:545–55. doi: 10.1056/nejmoa1510198 27509102

[23] Hughes DA Nicholls K Shankar SP Sunder-Plassmann G Koeller D Nedd K . Oral pharmacological chaperone migalastat compared with enzyme replacement therapy in Fabry disease: 18-month results from the randomised phase III ATTRACT study. J Med Genet. (2017) 54:288–96. doi: 10.1136/jmedgenet-2016-104178 27834756 PMC5502308

[24] Welford RWD Mühlemann A Garzotti M Rickert V Groenen PMA Morand O . Glucosylceramide synthase inhibition with lucerastat lowers globotriaosylceramide and lysosome staining in cultured fibroblasts from Fabry patients with different mutation types. Hum Mol Genet. (2018) 27:3392–403. doi: 10.1093/hmg/ddy248 29982630 PMC6140777

[25] Pushpakom S Iorio F Eyers PA Escott KJ Hopper S Wells A . Drug repurposing: progress, challenges and recommendations. Nat Rev Drug Discov. (2019) 18:41–58. doi: 10.1038/nrd.2018.168 30310233

[26] Sleire L Førde HE Netland IA Leiss L Skeie BS Enger P . Drug repurposing in cancer. Pharmacol Res. (2017) 124:74–91. doi: 10.1016/j.phrs.2017.07.013 28712971

