# Supplementary material for: GLA is associated with ESCC progression and chemotherapy response via DNA damage repair–related pathways
Source: Front Oncol. 2026 Jul 15;16:1900889. doi: 10.3389/fonc.2026.1900889 (PMC13414882; doi:10.3389/fonc.2026.1900889)
Supplement: Supplementary Table 1 — Experimental primer sequences. Name Sequence (5’-3’) [file Table1.docx]

Table S1. Experimental primer sequences.

Name Sequence (5’-3’)

| si-GLA-1# | sense:UCGUAGUAUCCAAAACUCCCATT |
| --- | --- |
|  | antisense:GGAGUUUUGGAUACUACGACATT |
| si-GLA-2# | sense:UACUUUUCCAGGAAUCAUCAATT |
|  | antisense:GAUGAUUCCUGGAAAAGUAUA TT |
| GLA | Forward:CTGAGGAACCCAGAACTACATCT |
|  | Reverse:GGTAGGCGTCCTTGCCAAT |
| GAPDH | Forward:TGTGGGCATCAATGGATTTGG |
|  | Reverse:ACACCATGTATTCCGGGTCAAT |
