# Supplementary figures and images for: GLA is associated with ESCC progression and chemotherapy response via DNA damage repair–related pathways
Source: Front Oncol. 2026 Jul 15;16:1900889. doi: 10.3389/fonc.2026.1900889 (PMC13414882; doi:10.3389/fonc.2026.1900889)

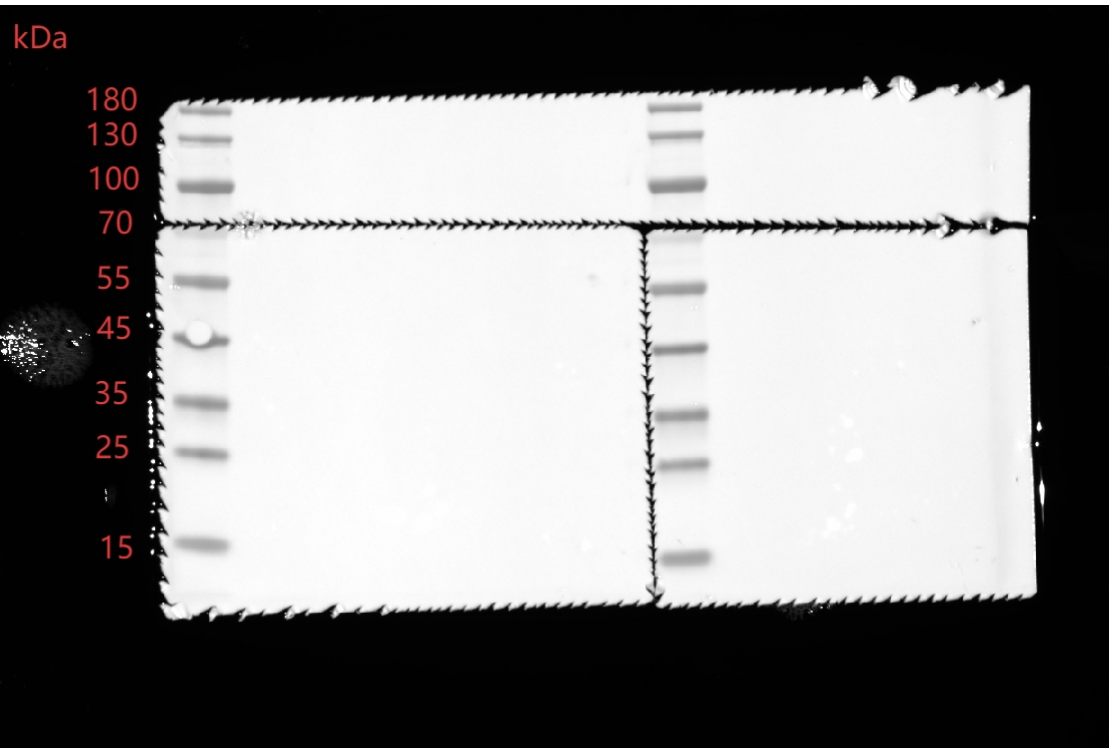

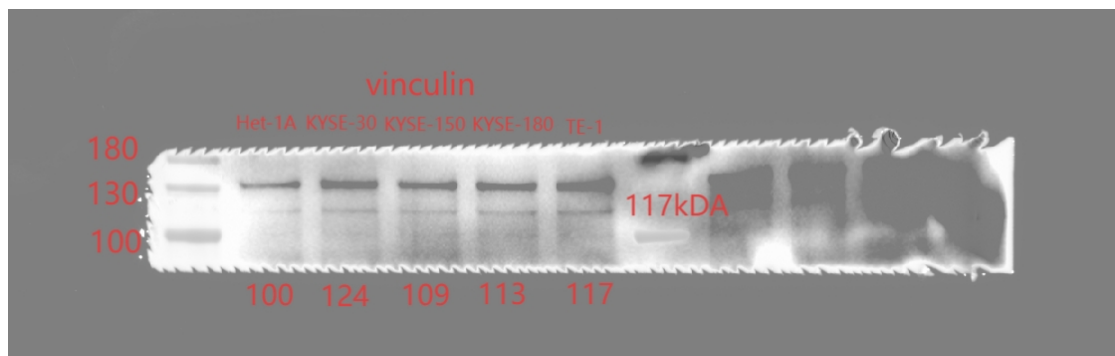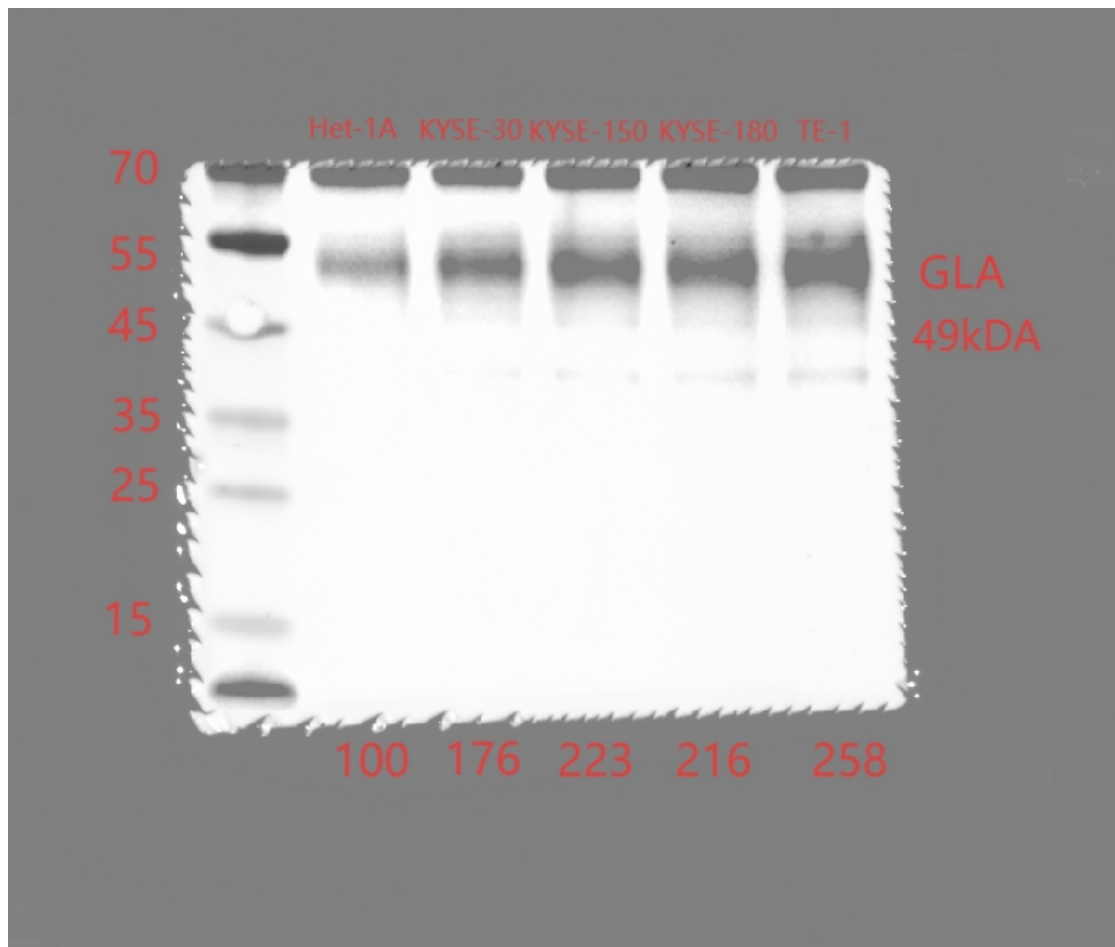

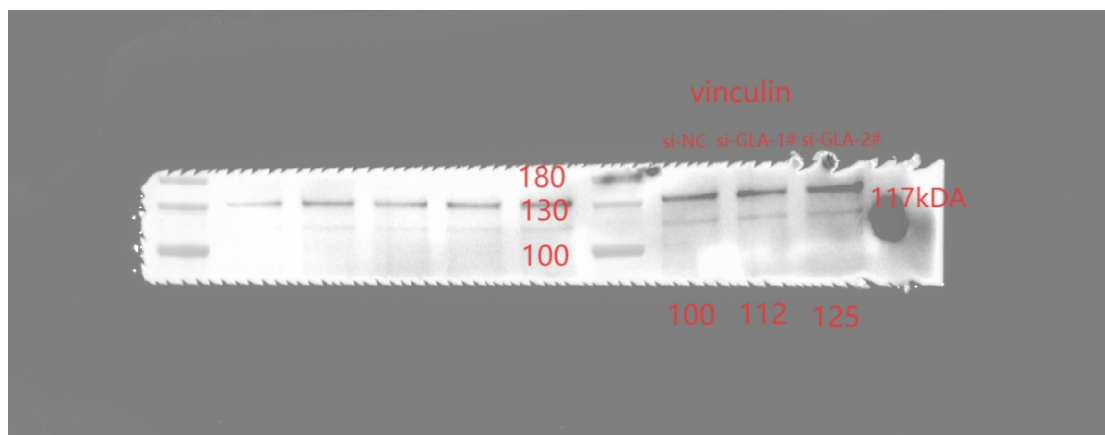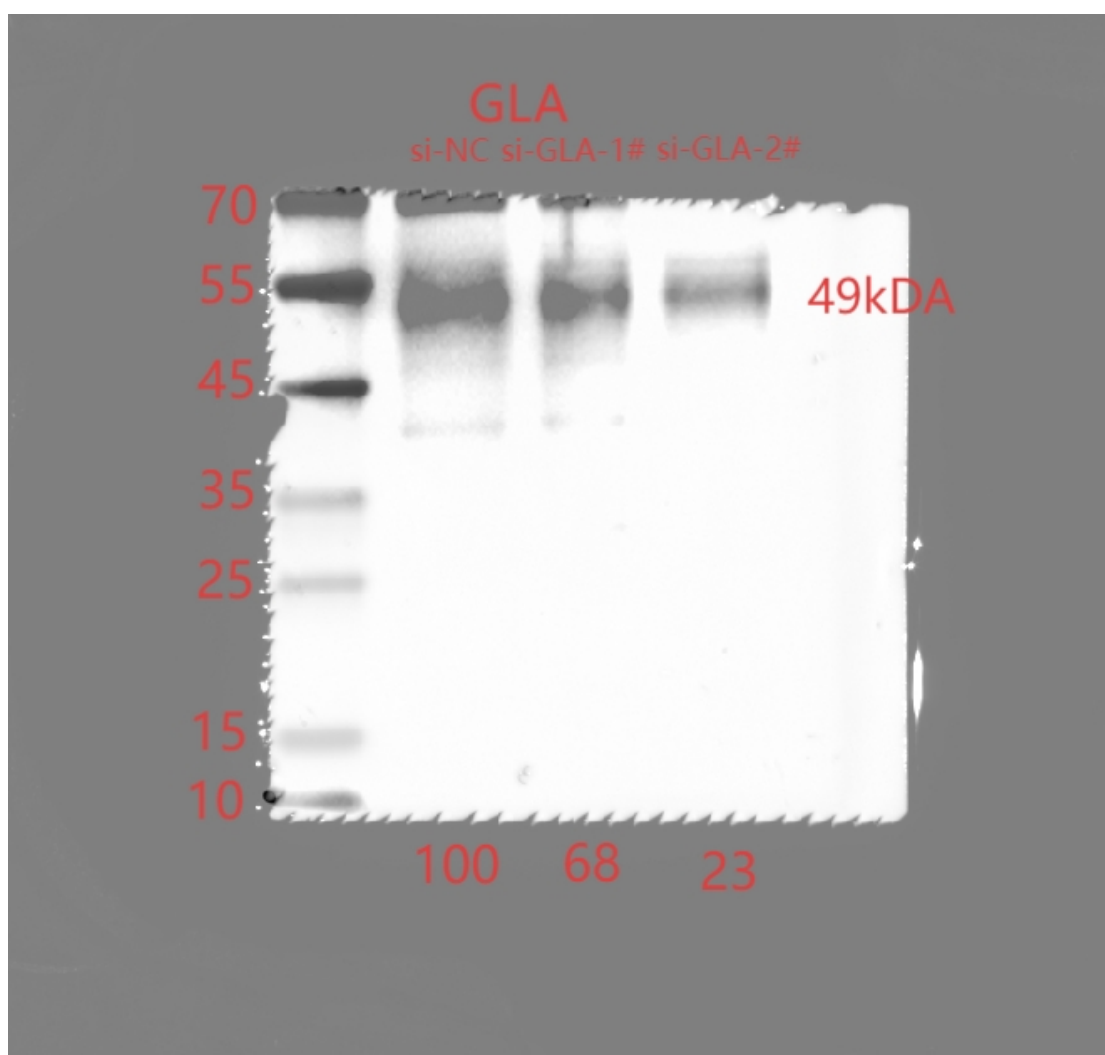

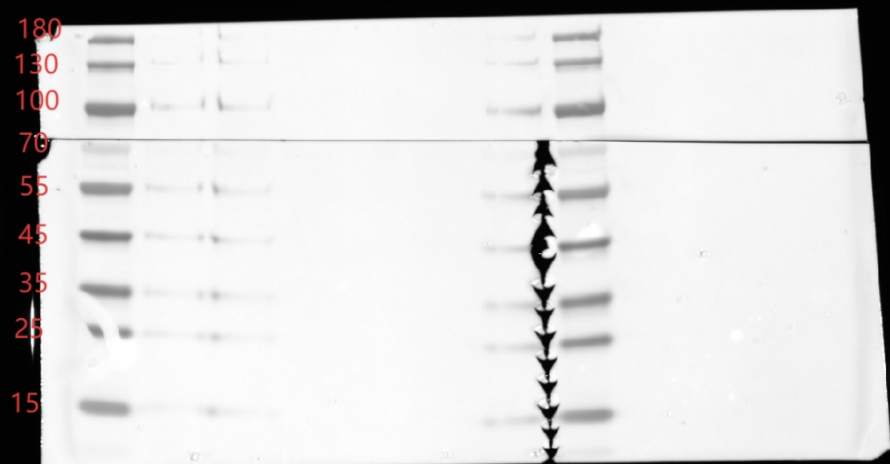

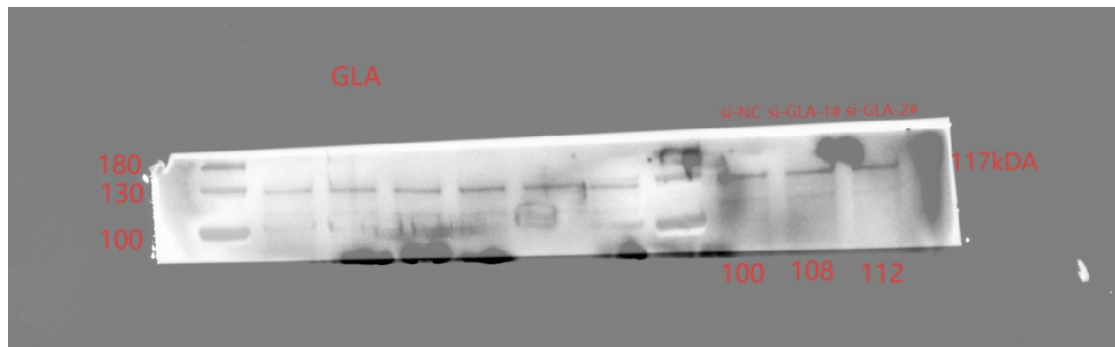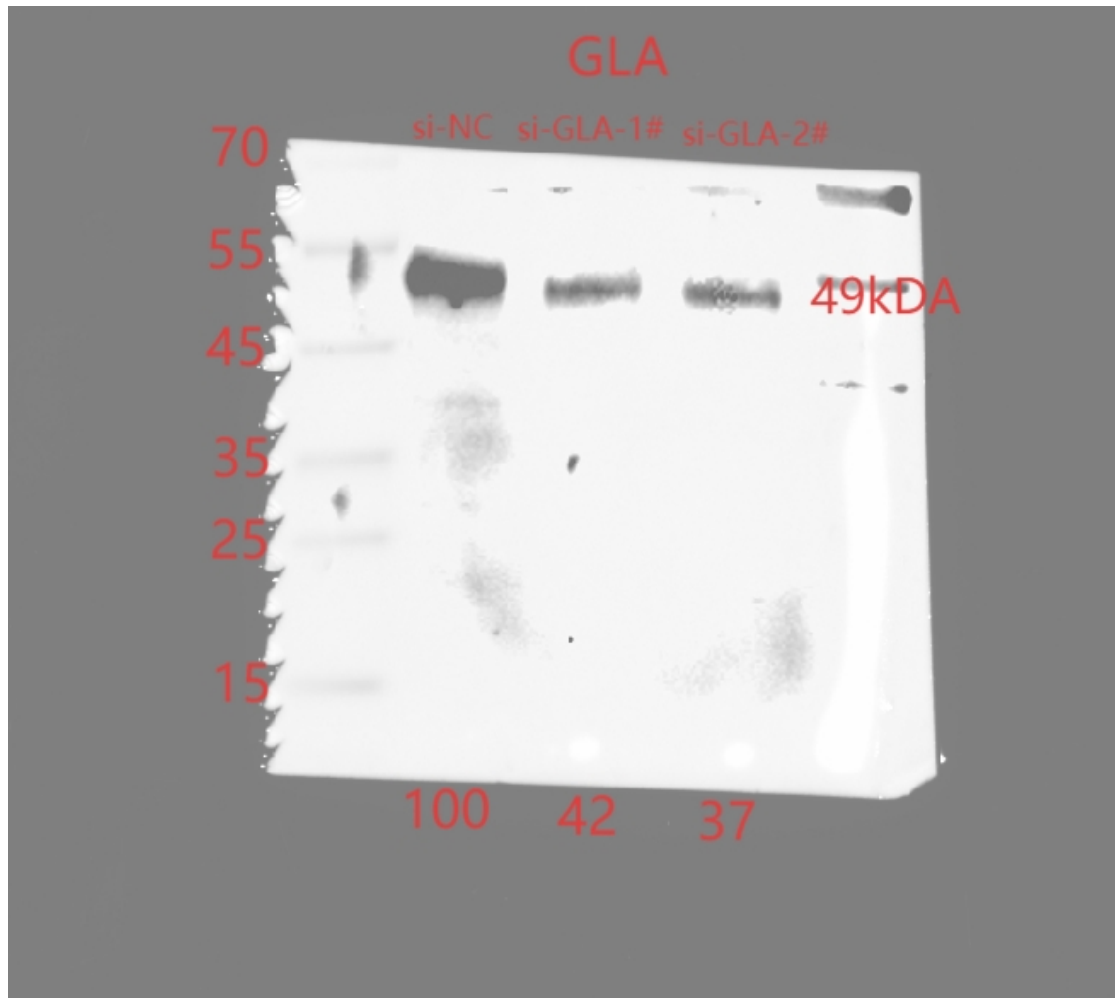

Supplement: Supplementary file 2 [file Image1.pdf]
